# Intravitreal Dexamethasone Implant in Autoimmune Retinopathy

**DOI:** 10.1155/2023/5670538

**Published:** 2023-03-31

**Authors:** Fernando Longhi Bordin, Carolina da Silva Mengue, Manuel Augusto Pereira Vilela

**Affiliations:** ^1^Institute Ivo Corrêa-Meyer, Porto Alegre, Brazil; ^2^Federal University of Health Sciences of Porto Alegre, Brazil

## Abstract

**Purpose:**

To describe the results of an intravitreal dexamethasone implant in managing autoimmune retinopathy (AIR).

**Methods:**

Two patients affected by AIR underwent intravitreal dexamethasone implantation and were followed by ocular coherence tomography, visual fields, and electroretinography.

**Results:**

The patients showed an interruption of the functional losses and remained stable with semestral injections.

**Conclusion:**

AIR is a complex condition with no standard treatment. The use of dexamethasone could be a valid option.

## 1. Introduction

First described in 1976 by Sawyer et al. [1], autoimmune retinopathy (AIR) is an underdiagnosed degenerative retinal disease of not fully understood physiopathology [2–7]. Vision loss is usually bilateral, asymmetric, painless, and progressive. Visual field loss is present with constrictions/scotomas, while photoreceptor loss promotes photosensibility, nyctalopia, and photopsia. The clinical examination may show some retinal pigmented epithelium lesions, vascular attenuation, and optic disc pallor with little or no intraocular inflammation [8, 9].

Signs and symptoms may precede cancer diagnosis. The lack of standard criteria makes diagnosis difficult. In the absence of clinically evident malignancy, a complete systemic workup should be done [2]. Antibody positivity is neither diagnostic nor pathognomonic, given that it can be also found in unaffected individuals. It is well known that autoimmune reaction is related to the presence of antiretinal antibodies—retinal cells. Antibodies against the retinal protein recoverin have the highest specificity and sensibility. Anti-alpha-enolase, antirod transducin, and anticarbonic anhydrase II are other retinal antibodies [3, 4]. Recently, the use of intravitreal dexamethasone implant has been described with a good response and with a decrease in the quantification of plasma autoantibodies [2].

The objective is to report cases of intravitreal dexamethasone implant in AIR and to discuss the existing evidence.

## 2. Case Report 1

**Figure 1:**
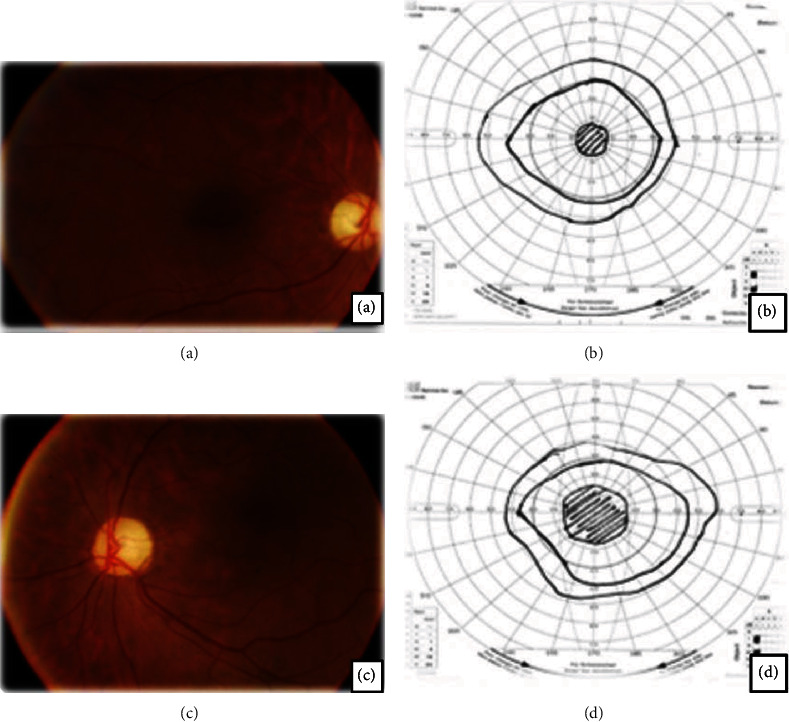
Retinography (a, c) showing optic disc pallor and arteriolar attenuation. The Goldmann visual field (b, d) showed bilateral constriction and central scotoma.

A 58-year-old white woman with diabetes type 2 (5-year duration) taking metformin 1 g/day came for evaluation due to progressive visual loss associated with visual field constriction and bilateral scotomas. Visual acuity was 20/40 bilateral (Snellen chart). Ishihara plates, ocular motility, applanation tonometry, pupillary reflex, and anterior biomicroscopy were normal. Retinal mapping showed light arterial narrowing, optic disc pallor, and focal RPE alterations. FA showed few microaneurysms in the left eye (LE). VF (Goldmann perimeter) showed bilateral and concentric visual field loss. OCT showed thinning of the photoreceptor layer and sparse disruption of the ellipsoid zone. ERG showed an accentuated reduction in photopic and scotopic responses and in implicit time. Serologic investigations (including syphilis, tuberculosis, and toxoplasmosis) were nonreactive. Cardiovascular, hematologic, and neurologic investigations were normal. Thorax tomography showed a tumoral lesion in the right pulmonary lobe. The patient was submitted for lobectomy. Antirecoverin became positive. Treatment was initiated with oral and subtenonian triamcinolone, without response. Systemic immunosuppression was not tolerated. Ozurdex has been injected at 6/6-month intervals (last 2 years), and no more changes were observed in comparative examinations. The patient has remained functionally stable (Figure 1).

## 3. Case Report 2

**Figure 2:**
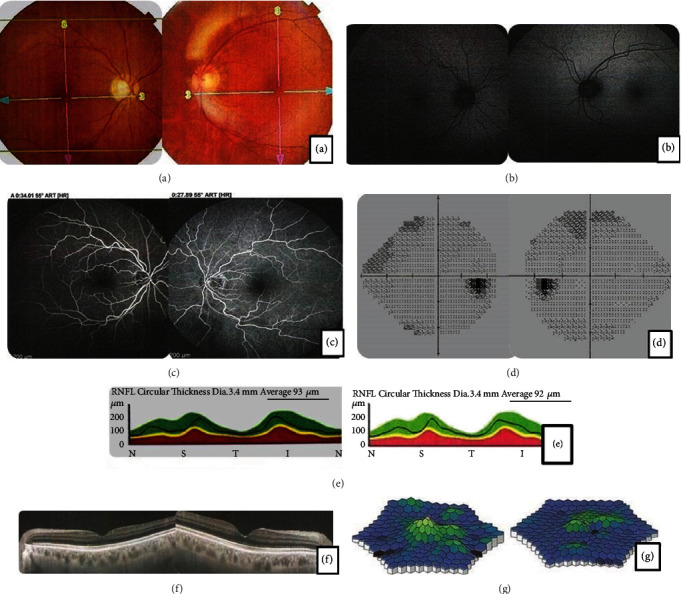
Retinography (a) and FA (c) showed important arterial narrowing. IVFA (b) showed no alterations. Humphrey visual field (d) showed diminished bilateral nasal sensitivity. Optic disc and macula OCT (e, f). ERG (g) showed diminished bilateral responses.

A 66-year-old caucasian male was referred complaining of bilateral, progressive, painless loss of visual acuity. The examination showed a visual acuity (Snellen) of 20/200 in the right eye (RE) and counting fingers in the left eye (LE). Normal external ocular motility with pupillary reflexes was preserved but slowed. Pseudophakic (intraocular lenses in the bag, no capsular or intraocular lens opacities). Retinal mapping showed normal optic discs, both maculas preserved, and diffuse arterial narrowing. One year and a half before, the patient was submitted to a total unilateral nephrectomy due to a renal tumor. Macula OCT showed no alterations. The visual field (Humphrey) showed diminished bilateral nasal sensitivity. FA showed significant arterial narrowing. ERG showed reduced bilateral responses. Diagnosis of presumed ca-AIR was established (in our country, there is no way to obtain an antibody dosage). Treatment begun with systemic immunosuppression, but the side effects were not well tolerated. Ozurdex was chosen and used at 6/6-month intervals. Since then, visual acuity has stayed at 20/200 in RE and oscillates from counting fingers to 20/400 in LE with discreet better ERG responses in RE and worsening in LE. There were no implicit time differences in both ERGs (Figure 2).

## 4. Discussion

AIR diagnostic is challenging and requires high suspicion level. Divided into two clinical forms, the more prevalent is nonneoplastic AIR (nnpAIR), and the other possibility is neoplastic AIR (subdivided into cancer-associated retinopathy (CAR) and melanoma-associated retinopathy (MAR)). Depending on the affected cells and the type of antibody, signs and symptoms may vary. CAR is associated with both cone and rod dysfunction, while MAR is associated with rod dysfunction. Systemic cancer and autoimmune diseases, even when it is not possible to evaluate antiretinal antibodies, associated with electroretinogram-specific abnormalities and progressive vision loss (symptomatic or not), may lead to suspicion of AIR. Antibody positivity should not be considered alone: clinical findings and multimodal imaging are fundamental. There is no imaging biomarker for this condition [2, 9].

Evidence shows that AIR may result from molecular mimicry between various retinal proteins and tumoral antigens with bacterial or viral proteins. Cytotoxic properties of the antiretinal antibodies lead to cell apoptosis. The dosage of antiretinal antibodies is scarcely available. This favors a presumed AIR diagnosis [2].

There are various therapeutic options described for AIR, but there is no standard treatment currently [8, 10–17]. When a diagnosis is established, treatment must be initiated aiming to achieve visual stabilization as this decision is focused on preventing disease progression [9]. Systemic immunosuppression may be auxiliary in retinal disease control and is the usual treatment of choice, though with several systemic side effects. Local treatment is an excellent option whenever possible [2].

Intravitreal or subtenonian triamcinolone could be initiated in association with systemic immunosuppression, whereby the latter seems to show the best results [18–20]. Vitamin supplementation, lutein, and antioxidants could be beneficial for retinal degeneration, but there is no strong evidence to recommend them. Plasmapheresis can also be used in the reduction of antiretinal antibodies and damage to the photoreceptor [11, 14, 21]. Tumor exeresis, associated or not with chemotherapy or radiation, seems not to impact the visual prognosis, as the antibodies are already circulating. Little visual recovery can happen, although functional stabilization is the most frequently observed response [2, 5].

An emerging treatment option refers to the use of a biodegradable intravitreal implant of dexamethasone (Ozurdex®, Allergan) [22–26]. As the implant gradually disintegrates, dexamethasone is slowly released within the vitreous over a 3- to 4-month period. Its pharmacokinetic drug profile is similar to the pharmacokinetics achieved with pulse administration of systemic corticosteroids, during which a short, initial, high-dose period is followed by a long, low-dose period. High concentrations of corticosteroid are associated with immunologic effect, like T cell apoptosis. Our patients did not tolerate systemic immunosuppression. This suggests that Ozurdex® may benefit patients with retinal diseases, such as uveitis, in which T cells play a central role in pathogenesis. First use of intravitreal dexamethasone is described in association with repeated doses of intravitreal triamcinolone acetonide with a positive response and an important decrease in plasma antirecoverin autoantibody [22].

All this evidence shows that Ozurdex® seems to be an excellent option with good tolerance, few side effects, and good disease control. Its mechanism seems to act by controlling local inflammation and recompounding hematoretinal barriers. Ultimately, intravitreal dexamethasone will be controlling local immunity, through T cell control [22–26].

Our patients have remained stable so far. We do not know however when and if it will be possible to interrupt the treatment. We have been using an extended way, and the criteria for reinjections are based on anatomical or functional changes. Unfortunately, access to autoantibody dosage is very difficult in our part of the world. To the best of our knowledge, few articles worldwide describe the use of Ozurdex® in RIA and without the use of other intravitreal corticosteroids. There is much more to discover and much more to know in order to understand the exact mechanism of Ozurdex® in AIR.

## Data Availability

The datasets used and/or analyzed during the current study are available from the corresponding author upon reasonable request.
